# Comparative efficacy, toxicity, and insulin-suppressive effects of simvastatin and pravastatin in fatty acid-challenged mouse insulinoma MIN6 β-cell model

**DOI:** 10.3389/fendo.2024.1383448

**Published:** 2024-10-31

**Authors:** Hossein Arefanian, Sardar Sindhu, Fatema Al-Rashed, Fawaz Alzaid, Ashraf Al Madhoun, Mohammed Qaddoumi, Fatemah Bahman, Michayla R. Williams, Shaima Albeloushi, Nourah Almansour, Rasheed Ahmad, Fahd Al-Mulla

**Affiliations:** ^1^ Immunology and Microbiology Department, Dasman Diabetes Institute, Kuwait City, Kuwait; ^2^ Animal and Imaging Core Facility, Dasman Diabetes Institute, Kuwait City, Kuwait; ^3^ Department of Bioenergetics and Neurometabolism, Dasman Diabetes Institute, Kuwait City, Kuwait; ^4^ Institut Necker Enfants Malades (INEM), French Institute of Health and Medical Research (INSERM), Immunity and Metabolism of Diabetes (IMMEDIAB), Université de Paris Cité, Paris, France; ^5^ Department of Genetics and Bioinformatics, Dasman Diabetes Institute, Kuwait City, Kuwait; ^6^ Department of Biochemistry and Molecular Biology, Dasman Diabetes Institute, Kuwait City, Kuwait; ^7^ Department of Pharmacology and Therapeutics, College of Pharmacy, Kuwait University, Kuwait City, Kuwait

**Keywords:** pancreatic β-cells, MIN6 cells, statin, simvastatin, pravastatin, mitochondrial respiration

## Abstract

**Introduction:**

Familial hypercholesterolemia, the highly prevalent form of dyslipidemia, is a well-known risk factor for premature heart disease and stroke worldwide. Statins, which inhibit 3-hydroxy 3-methylglutaryl coenzyme A (HMG-CoA) reductase, are the first-choice treatment for dyslipidemias, and have been effective in reducing the risk of stroke and myocardial infarction. However, emerging evidence indicates that statins may increase the incidence of new-onset type 2 diabetes by reducing β-cell mass and function. Notably, past *in vitro* reports studying the effects of statins on β-cells were performed without including free fatty acids in the model. This factor should have been addressed since these agents are used to treat individuals with hyperlipidemia.

**Methods:**

Here, we used a mouse insulinoma MIN6 β-cell culture model to assess the efficacy, cytotoxicity, and insulin-suppressive effects of simvastatin and pravastatin in the presence of palmitic, linoleic, and oleic acids cocktail to mimic mixed lipids challenge in a biologically relevant setting.

**Results and discussion:**

Our findings indicate that simvastatin was more effective in lowering intracellular cholesterol but was more cytotoxic as compared to pravastatin. Similarly, simvastatin exhibited a higher suppression of total insulin content and insulin secretion. Both drugs suppressed insulin secretion in phases 1 and 2, dose-dependently. No significant effect was observed on mitochondrial respiration. More importantly, elution experiments showed that insulin content diminution by simvastatin treatment was reversible, while exogenous mevalonate did not improve total insulin content. This suggests that simvastatin's influence on insulin content is independent of its specific inhibitory action on HMG-CoA reductase. In conclusion, our study identified that simvastatin was more effective in lowering intracellular cholesterol, albeit it was more toxic and suppressive of β-cells function. Notably, this suppression was found to be reversible.

## 1 Introduction

Familial hypercholesterolemia, characterized clinically by elevated levels of low-density lipoprotein (LDL) cholesterol, is a highly prevalent form of dyslipidemia that is associated with premature cardiovascular disease development (1). Lipid-lowering drugs such as statins, which act as hydroxymethyl glutaryl coenzyme A (HMG-CoA) reductase inhibitors, decrease the vascular-related diseases including stroke and myocardial infarction and reduce mortality by about 10% for every 1.0 mmol/L reduction achieved in LDL-cholesterol levels (2). Statins, which have been in use for almost 50 years, are widely recommended as the first-line treatment of dyslipidemia, as per the guidelines of the European Society of Cardiology and the American Heart Association (3–5).

Statins are classified according to their solubility as hydrophilic statins (pravastatin and rosuvastatin) and lipophilic statins (atorvastatin, cerivastatin, fluvastatin, lovastatin, pitavastatin and simvastatin) based on the substituents on the ring attached to the active moiety. Although the target of both types of statins is HMG-CoA reductase, the inhibitory mechanisms are distinct (6, 7).

Despite that the safety and relative tolerability of statins are proven by several studies including observational studies (8–13), clinical trials (14, 15) and meta-analyses (16–22), emerging evidence indicates that statins can increase the onset of type 2 diabetes (T2D) due to a reduction in insulin sensitivity and a decrease in β-cells mass and function in a dose-dependent manner. It has been noted that lipophilic statins are generally more diabetogenic compared to hydrophilic statins (16, 17). According to the JUPITER trial, treatment with high statin concentrations resulted in a further increase the rate of new-onset T2D by about 10% (22, 23). While the mechanism by which statins lower the lipids is relatively well understood, the mechanism(s) underlying the induction of type 2 diabetes appear(s) to be multifactorial and elusive.

In a recent animal study, β-cell specific HMG-CoA reductase KO mice were found to develop severe hypoinsulinemic hyperglycemia, primarily due to defective β-cell development, maturation, and proliferation postnatally, and β-cell mass reduction. Furthermore, it was speculated that trans-differentiation of β-cells to α-cells at perinatal period could also be involved in the loss of β-cells mass. Regarding functional responses, glucose-stimulated insulin secretion (GSIS) was also found to be impaired in pancreatic islets from these mice (24).

With regard to *in vitro* investigations, several studies using the MIN6 mouse (25–30) and INS-1 rat insulinoma cell models (31–33) treated with statins have uncovered the associated mechanisms of impairment of β-cell survival and function. These studies demonstrated that statins induced apoptosis as well as impairment of β-cell function by direct inhibition of ATP-sensitive potassium (K-ATP) channels in a mitochondria-independent manner and by interfering with mitochondrial respiration, thus decreasing cytosolic ATP levels, and inhibiting metabolic upregulation of L-type Ca^2+^ channels. These studies concluded that these inhibitory effects on K-ATP channels were more likely related to lipophilic statins, such as simvastatin, rather than hydrophilic statins, such as pravastatin (25, 27, 31, 34).

It is important to note that the past *in vitro* studies investigating statins’ effects on β-cells were conducted without the presence of free fatty acids in the *in vitro* cell model which should have been addressed, given that these statins are indicated to treat patients with dyslipidemia. Therefore, we herein used a mouse insulinoma MIN6 β-cell culture model to compare the efficacy, cytotoxicity, and insulin-suppressive effects of simvastatin and pravastatin, in the presence of palmitic, linoleic, and oleic acids, to mimic effects of mixed lipids challenge in a biologically relevant setting.

## 2 Materials and methods

### 2.1 Cell culture and treatments

MIN6 cells, a mouse insulinoma β-cell line, were a generous gift from Dr. Jun-ichi Miyazaki, Kumamoto University Medical School, Japan (35). The cells were routinely maintained in Dulbecco’s modified Eagle’s medium (DMEM), which contained 25.2 mM glucose, supplemented with 15% heat-inactivated fetal bovine serum (FBS), 2 mM l-glutamine, 1 mM sodium pyruvate, 10 mM HEPES, 50 μg/mL penicillin, 100 μg/mL streptomycin, and 70 mM β-mercaptoethanol at 37°C, with 5% CO_2_ (all reagents were purchased from Invitrogen, Waltham, MA, USA). The cells were re-fed every 2 to 3 days (35). For experimental assays, the cells were maintained in the supplemented DMEM containing 5.6 mM glucose. All analyses presented in this study on MIN6 cells were performed at a passage of <25.

A stock solution (100 mM) of simvastatin (ab120505, Abcam, Waltham, MA, USA) and pravastatin sodium salt (ab142617, Abcam, Waltham, MA, USA) were freshly prepared in 100% ethanol and phosphate-buffered saline (PBS), respectively. The cells were treated with simvastatin or pravastatin at different concentrations, depending on each experimental setting. Stock solution (100 mM) of mevalonolactone (M4667, Sigma-Aldrich, St. Louis, MO, USA) was freshly prepared in 95% ethanol.

### 2.2 Preparation of palmitic, linoleic, and oleic acids cocktail

A lipid cocktail containing palmitic acid, linoleic acid, and oleic acid conjugated with fatty acid-free bovine serum albumin (BSA) was prepared, as described (36, 37). Briefly, a 24% (w/v) BSA solution (Cat #: A6003; Sigma-Aldrich, St. Louis, MO, USA) was prepared in a 150 mM NaCl solution (pH 7.4), filtered (0.45μm) and aliquoted. To prepare the PLO cocktail, first a stock solution of palmitic acid, linoleic caid, and sodium oleate, was made freshly in ethanol (0.5 mL) which was later evaporated under a gentle stream of nitrogen. Each fatty acid stock solution was mixed with ice cold 24% (w/v) BSA solution to yield a final concentration of 10 mM, each. Fatty acid mixtures containing BSA were heated gently and stirred on a hot plate to yield a uniform emulsion which can be stored at -20°C until use. At the final step, 10 mM stock solution each of palmitic acid, linoleic acid, and sodium oleate was mixed in a 4:3:3 ration, respectively. All chemicals were purchased from Sigma-Aldrich (St. Louis, MO, USA).

### 2.3 Cell survival assay

Cells (5×10^4^ cells/well) were seeded in 96-well plates (Costar, High Wycombe, UK). Two days later, the cells were treated with different concentrations of simvastatin, pravastatin, and/or PLO for 24, 48, or 72 hrs, following which, cell survival was determined using an MTT (3-[4,5-dimethylthiazol-2-yl]-2,5 diphenyl tetrazolium bromide) assay (Trevigen, Minneapolis, MN, USA), according to the manufacturer’s instructions. The plates were analyzed using the Synergy H4 Hybrid Microplate Reader (BioTek, Winooski, VT, USA) and data analysis was performed using Gen5 software ver 2.03. Data are expressed as % viability to that of untreated or vehicle treated as control.

### 2.4 Total cholesterol content assay

The intracellular level of total cholesterol content was measured using cell-based cholesterol assay kit (ab133116, Abcam, Waltham, MA, USA). Briefly, 3×10^4^ cells/well were seeded in a 96-well plate. A day after, cells were treated with different concentrations of simvastatin or pravastatin alone or in combination with PLO for 72 hrs. Then the level of total cholesterol content was measured based on the kit instruction by measuring the level of fluorescent intensity (excitation at 340-380 nm/emission at 385-470 nm) using the Synergy H4 Hybrid Microplate Reader (BioTek, Winooski, VT, USA). Data analysis was performed data using Gen5 software ver 2.03.

### 2.5 Total insulin content and GSIS assays

To measure the total insulin content of the cells, 1×10^5^ cells/well were grown, in 24-well plates (Costar, High Wycombe, UK). After 24 hrs, the cells were treated with different concentrations of simvastatin or pravastatin, alone or in combination with PLO, for another 72 hrs. Then the medium was removed, cells were washed twice with PBS and cells were incubated for 1 hr with 500 μl of 2.8 mM glucose supplemented in Krebs–Ringer HEPES buffer (basal media, pH-adjusted to 7.4 with 1 mol/l NaOH), containing 135 mM NaCl, 3.6 mM KCl, 5 mM NaHCO_3_, 0.5 mM MgCl_2_, 1.5 mM CaCl_2_, 10 mM HEPES, and 0.1% BSA (Sigma-Aldrich, St. Louis, MO, USA). Then, 500 μl of cold acidified ethanol (1.5% HCl in 70% EtOH) was added to each well after removing the Krebs-Ringer HEPES buffer and washing with PBS. Cells were kept overnight at -80°C. The following day, three freeze/thaw cycles (-20°C/4°C) were performed to lyse the cells. The lysed cells were removed by scraping and were transferred to labeled tubes and the tubes were centrifuged for 15 min at 15000×g at 4°C. The supernatants were transferred to new labeled tubes, acid-ethanol extracts were neutralized with same volume of 1M Tris pH 7.5 and were kept in -20°C for insulin measurements using ultrasensitive mouse insulin ELISA kit (Mercordia, Sylveniusgatan, Uppsala, Sweden).

GSIS assay was performed using the static incubation method (38). Briefly, 1×10^5^ cells/well were grown in 24-well plates (Costar, High Wycombe, UK). After 24 hours, cells were treated with different concentrations of simvastatin or pravastatin, alone or in combination with PLO, for another 72 hrs. Then the medium was removed, and the cells were washed twice with PBS. Next, for equilibration, cells were incubated for 1 hr with 500 μl of 2.8 mM glucose, supplemented in Krebs–Ringer HEPES buffer. After equilibration, 500 μl of glucose-containing stimulation media, Krebs–Ringer HEPES buffer supplemented with 2.8, 5.6, or 16.8 mM glucose or Krebs-Ringer HEPES buffer supplemented with KCl (35 mM, adjusted osmolarity by decreasing NaCl level) was added to each well and incubated for 2 hrs at 37°C. Following which, the GSIS assay was terminated by placing the plate on ice. Conditioned medium (500 μl) was removed from each well, centrifuged at 300×g at 4°C for 5 min, transferred to an ice-cold Eppendorf tube, and kept in -20°C to detect the level of secreted insulin using ultrasensitive mouse insulin ELISA kit (Mercordia, Sylveniusgatan, Uppsala, Sweden).

To measure the level of secreted insulin during phase 1, all steps mentioned above for GSIS were followed except the last step in which the GSIS assay was stopped at 15 min and then conditioned medium was removed from each well and processed for measuring the level of insulin.

To measure the level of secreted insulin produced during phase 2, after the step of treating cells with simvastatin or pravastatin, cells were treated with Krebs–Ringer HEPES buffer supplemented with KCl (35 mM) for 20 min at 37°C, followed by washing twice with PBS, then 500 μl of Krebs–Ringer HEPES buffer supplemented with 2.8, 5.6, or 16.8 mM glucose was added to each well and incubated for 1 hr at 37°C, then the conditioned medium was removed from each well and processed for measuring the level of insulin.

The levels of total insulin content and secreted insulin were normalized by the level of total protein measured by Bradford assay (Bio-Rad, Hercules, California, USA) against a standard curve.

### 2.6 Metabolic flux analysis (Seahorse assay)

Oxygen consumption rate (OCR) was analyzed using the manufacturer’s protocol for Mitochondrial Stress Test on a metabolic flux analyzer (Seahorse XFe96, Agilent Technologies, Santa Clara, California, USA). MIN6 cells were seeded in Agilent Seahorse XF96 Cell Culture Microplate (Cat#: 101085-004) at 4×10^4^ cells/well for 24 hrs, after which, the cells were either treated with 100 nM, 1 μM, and 10 μM concentrations of simvastatin and pravastatin, alone or in combination with PLO (500 μM), for 72 hrs or were left untreated (treated with vehicle only). At the end point of each condition, OCR assay was performed using compounds that were prepared in assay media supplemented with 5.6 mM glucose at the following concentrations: Oligomycin (1 µM), Carbonyl cyanide 4-(trifluoromethoxy)phenylhydrazone (FCCP; 2 µM), and Rotenone/Antimycin A (0.5 µM) from Agilent Technologies Seahorse XF Cell Mito Stress Test Kit (Cat# 103015-100, Agilent Technologies, Santa Clara, California, USA). The levels of basal and maximal respirations were calculated based on the kit’s instructions and OCR was normalized by the number of cells (39).

### 2.7 Statistical analysis

The data are expressed as Mean ± SEM of at least three independent experiments, and in each experiment, three replicates were processed with similar results. Group differences were analyzed using the unpaired Student’s t-test and one-/two-way Analysis of Variance (ANOVA) test, followed by Tukey’s multiple comparisons test using GraphPad Prism 9.0 (GraphPad Software, CA, USA). P-values < 0.05 were considered statistically significant, and the statistical significance was expressed as *P<0.05, **P<0.01, ***P<0.001, or ****P<0.0001.

## 3 Results

### 3.1 Toxicity and cholesterol lowering effects of simvastatin and pravastatin

**Figure 1:**
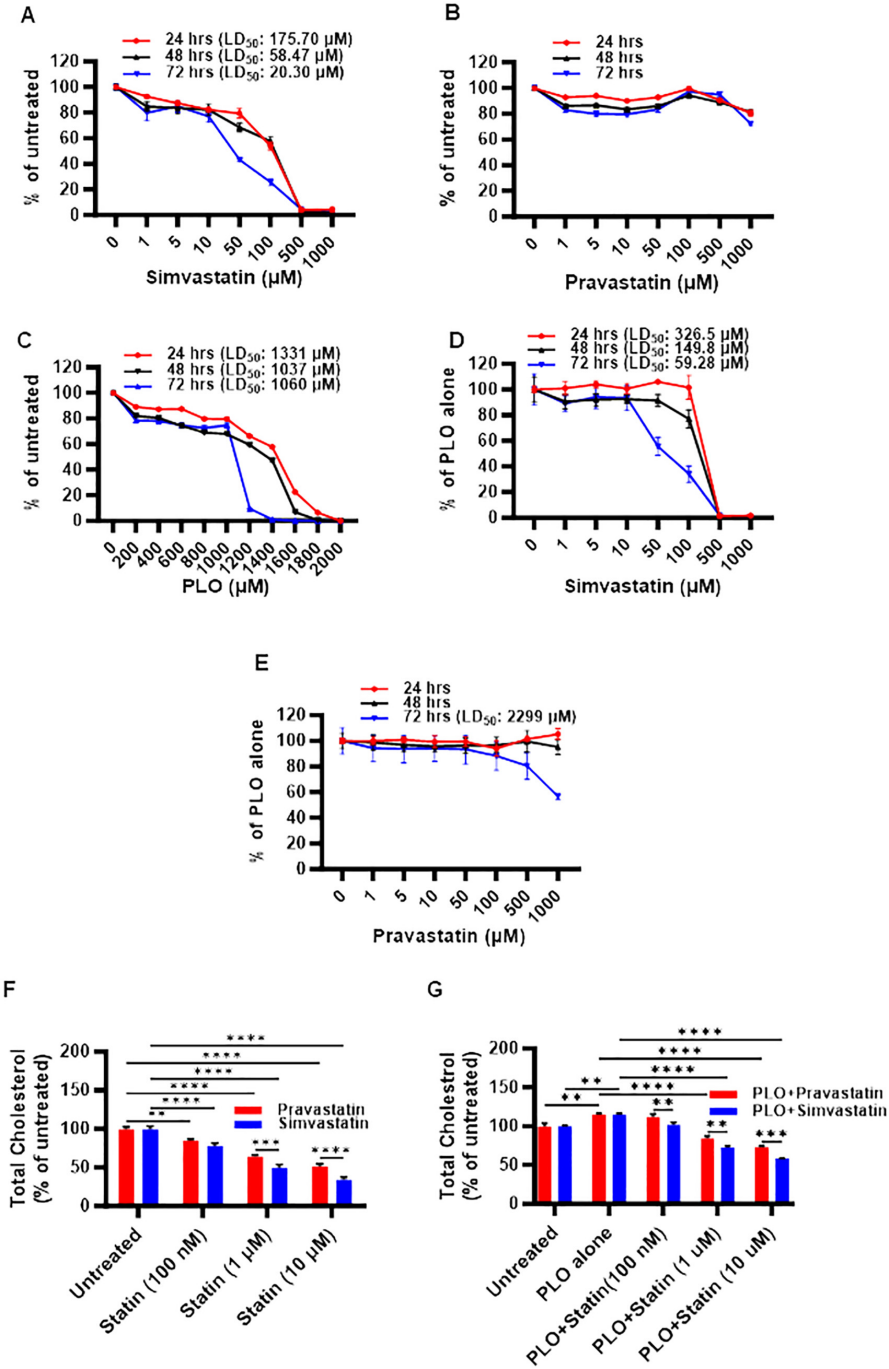
Toxicity and cholesterol lowering effects of simvastatin and pravastatin in mouse insulinoma MIN6 cells. MIN6 cells were maintained in supplemented DMEM media with 5.6 mM glucose and were treated with different concentrations of either **(A)** simvastatin or **(B)** pravastatin (0, 1, 5, 10, 50, 100, 500, and 1000 μM) or **(C)** fatty acid cocktail containing palmitic, linoleic, and oleic acids (PLO; 0, 200, 400, 600, 800, 1000, 1200, 1400, 1600, 1800, and 2000 μM) for 24 hrs (red line), 48 hrs (black line) and 72 hrs (blue line). In modified MIN6-PLO set-up, MIN6 cells cultured in 5.6 mM glucose were treated with a combination of PLO (500 μM) and either **(D)** simvastatin or **(E)** pravastatin (0, 1, 5, 10, 50, 100, 500, and 1000 μM) for 24 hrs (red line), 48 hrs (black line) and 72 hrs. MTT assay was conducted to measure the lethal dose of 50% survival (LD_50_). The level of total intracellular cholesterol contents were determined in MIN6 cells in DMEM containing 5.6 mM glucose treated (100 nM, 1 and 10 μM) with **(F)** simvastatin or pravastatin alone, and **(G)** in combination with PLO (500 μM). **(F)** Vehicle-treated cells served as untreated control (Red bar for ethanol as simvastatin vehicle, and Blue bar for PBS as pravastatin vehicle). **(G)** Vehicle-treated cells served as untreated control (Red bar for a combination of ethanol and BSA as simvastatin and PLO vehicles, and Blue bar for a combination of BSA, ethanol and PBS as pravastatin and PLO vehicles). Data are presented as mean ± SEM values (n = 3 independent experiments, each experiment was done in triplicate wells) and were analyzed using one- or two-way ANOVA multiple comparisons test. **p < 0.01; ***p <0.001; and ****p <0.0001.

First, we investigated the impact of simvastatin and pravastatin on the viability of MIN6 cells, and since this cell model involved lipotoxic exposure to PLO, we determined toxicity effects on MIN6 cells both in absence and presence of PLO. To this effect, MTT assay performed at 24, 48, and 72 hrs revealed that cell viability decreased at all time points with increasing concentrations of simvastatin and PLO, but not with pravastatin, whereas no changes were observed regarding respective vehicle controls (**Figure 1**; **Supplementary Figure S1**). Regarding simvastatin alone, the calculated lethal doses of 50% (LD_50_) were 175.70, 58.47, and 20.30 μM at 24, 48, and 72 -hrs, respectively (**Figure 1A**). Importantly, no notable toxicity effect on MIN6 cells were observed with pravastatin alone treatment at these time points (**Figure 1B**). Similarly, PLO treatment alone induced toxicity in MIN6 cells at concentrations of 1331, 1037, and 1060 μM at 24, 48, and 72 hrs, respectively (**Figure 1C**). Simvastatin plus PLO combination treatment induced cell toxicity (LD_50_) at concentrations of 326.5, 149.8, and 59.28 μM at 24, 48, and 72 hrs, respectively (**Figure 1D**), indicating overall an improvement in cell viability at all time points compared to simvastatin alone (**Figure 1A**). Pravastatin plus PLO combination treatment induced cell toxicity only at LD_50_ of 2299 μM at 72 hrs, whereas no toxicity was observed at 24 and 48 hrs (**Figure 1E**). These findings further guided the next experiments for using either simvastatin or pravastatin at the concentrations of 100 nM, 1 μM and 10 μM, either alone or in combination with PLO (500 μM) for 72 hrs. Of note, at these selected concentrations, the cell viability was >80%, compared with respective controls i.e. cells treated with relevant vehicle only.

Next, we assessed the functional outcome of simvastatin and pravastatin treatments in MIN6 cells, alone or in combination with PLO. To this effect, both simvastatin and pravastatin alone were found to significantly reduce (p<0.01) the level of total cholesterol content in MIN6 cells at 72 hrs in a dose-dependent manner, compared with relevant vehicle-treated controls (**Figure 1F**). At all concentrations, except at 100 nM, a significant reduction of total cholesterol (p<0.001) was observed for simvastatin over pravastatin (**Figure 1F**). Adding PLO to cell culture media for 72 hrs significantly increased the total cholesterol content in MIN6 cells, compared to control, vehicle treated cells (**Figure 1G**, p<0.01). In this combination treatment model, the total intracellular cholesterol with simvastatin plus PLO or pravastatin plus PLO treatment was significantly less, compared to control (PLO alone) (p<0.0001). These reductions in total cholesterol were dose-dependent, showing a significant efficacy of simvastatin over pravastatin for lowering total cholesterol content in MIN6 cells at the three doses used (**Figure 1G**, p<0.01).

### 3.2 Effect of simvastatin and pravastatin treatments on total insulin content and GSIS

**Figure 2:**
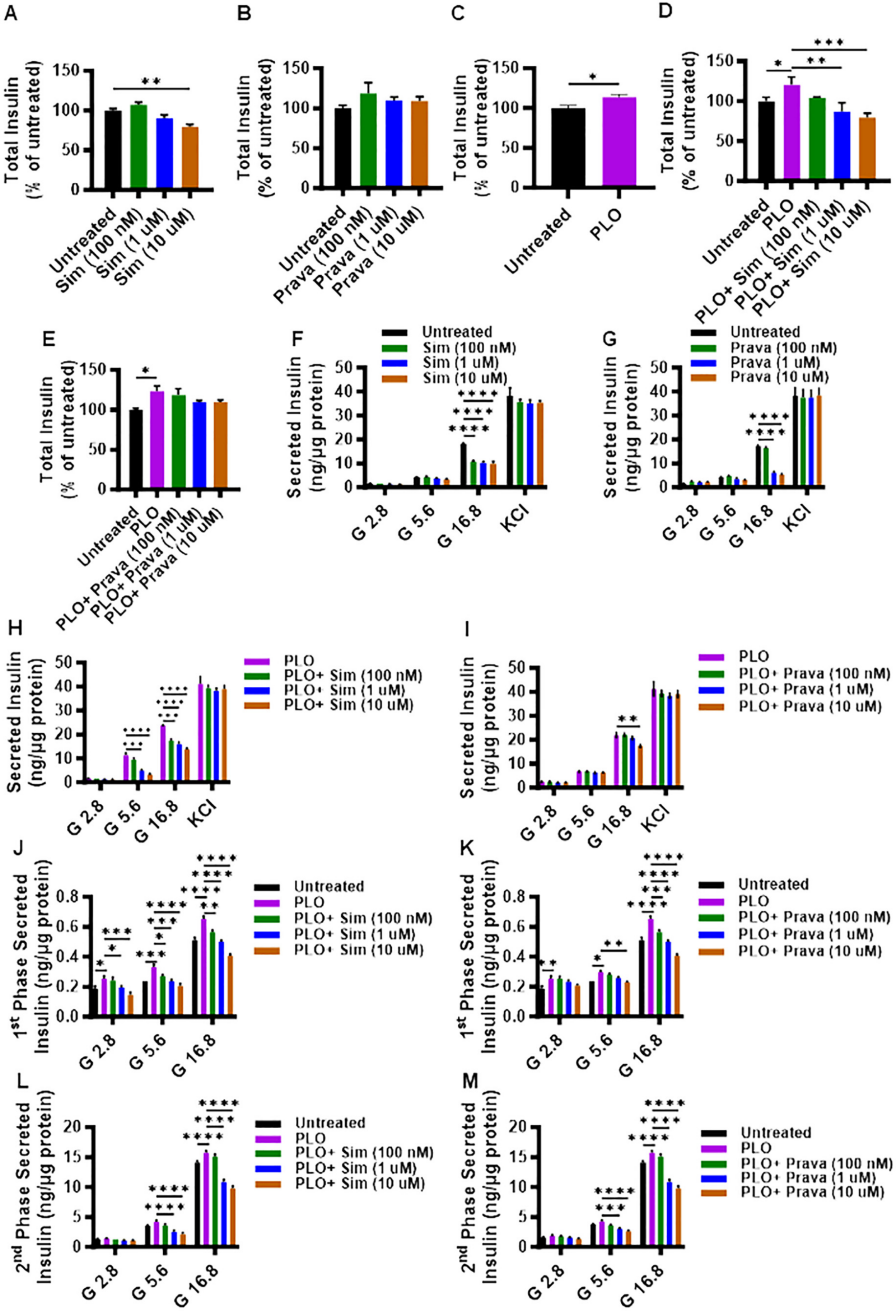
Effect of simvastatin and pravastatin treatments on total insulin content and glucose-stimulated insulin secretion (GSIS). MIN6 cells were maintained in DMEM media supplemented with 5.6 mM glucose treated with different concentrations (100 nM; dark green color bars, 1 μM; blue color bars, and 10 μM; brown color bars) of simvastatin or pravastatin alone or in combination with PLO (500 μM, purple color bars) for 72 hrs, and vehicle-treated cells served as untreated control (Black color bars). Total insulin content, shown as percentage of ratio of total insulin content in each condition relative to the level in untreated cells, were measured as described in Material and Methods. Total insulin content is shown in MIN6 cells treated with **(A)** simvastatin alone, **(B)** pravastatin alone, **(C)** PLO alone, **(D)** simvastatin plus PLO, and **(E)** pravastatin plus PLO. Total normalized secreted insulin, as measured by GSIS assay, is shown for MIN6 cells treated with **(F)** simvastatin alone, **(G)** pravastatin alone, **(H)** simvastatin plus PLO, and **(I)** pravastatin plus PLO. Level of secreted insulin during phase 1 (at 15 min incubation) is shown for MIN6 cells treated with a combination of **(J)** simvastatin plus PLO, and **(K)** pravastatin plus PLO. Level of secreted insulin during phase 2 (at 1 hr glucose challenge, following KCl treatment) is shown for MIN6 cells treated with a combination of **(L)** simvastatin plus PLO, and **(M)** pravastatin plus PLO. Each experiment was performed in triplicate. Group differences were analyzed using one-/two-way analysis of variance (ANOVA) and p-values < 0.05 were considered statistically significant. The representative data from three independent experiments with similar results are presented as Mean ± SEM values, and each experiments was performed in triplicate.*p < 0.05; **p < 0.01; ***p <0.001; and ****p <0.0001.

First, we investigated whether the total insulin content was modulated in MIN6 cells, following treatment for 72 hrs with either simvastatin or pravastatin. Compared to control, a significant reduction in the total insulin content was observed for simvastatin treatment, only at a higher concentration of 10 μM (**Figure 2A**, p< 0.01), whereas no significant differences in total insulin content were detected for pravastatin (**Figure 2B**). On the other hand, PLO treatment alone induced significantly higher total insulin content at 72 hrs (**Figure 2C**, p<0.05). In combination treatment model, the total insulin content of MIN6 cells treated with simvastatin and PLO was significantly less, compared to that of control (PLO alone) at 1 μM and 10 μM concentrations (**Figure 2D**, p<0.01). Notably, pravastatin plus PLO treatment had no significant effect on total insulin content of MIN6 cells under similar conditions (**Figure 2E**).

To further examine the effects of simvastatin and pravastatin on insulin secretion, GSIS was performed using static incubation method, with or without PLO. In the absence of PLO, only under condition of high glucose challenge (16.8 mM), a significant reduction in the secreted insulin was observed in MIN6 cells that were treated with different concentrations of either simvastatin (**Figure 2F**, p<0.0001) or pravastatin (except at 100 nM) (**Figure 2G**, p<0.0001). Moreover, no significant differences in secreted insulin levels were observed in response to glucose independent KCl challenge of MIN6 cells treated with different concentrations of either simvastatin or pravastatin (**Figure 2F**).

In presence of PLO, simvastatin treatments induced a significant reduction in secreted insulin by MIN6 cells challenged with 5.6 mM or 16.8 mM of glucose. However, a non-significant effect on secreted insulin was observed in response to 5.6 mM of glucose challenge in MIN6 cells treated with 100 nM of simvastatin, compared to control (PLO alone) (**Figure 2H**). Different concentrations of pravastatin plus PLO did not show significant effect on secreted insulin, except when used at the highest concentration of 10 μM, in response to 16.8 mM of glucose challenge (**Figure 2I**).

To further dissect the GSIS response in MIN6 cells that were treated with simvastatin in combination with PLO, the levels of secreted insulin during phase 1 and phase 2 of insulin secretion were evaluated separately. During phase 1 of insulin secretion, a significant increase in secreted insulin level was detected in cells treated with PLO alone, compared to control, when the cells were challenged with 2.8, 5.6, and 16.8 mM glucose (**Figures 2J, K**). Nonetheless, a combined treatment with simvastatin and PLO significantly decreased the secreted insulin in a dose-dependent manner, at the used three glucose concentrations (2.8, 5.6, and 16.8 mM), compared to PLO alone treated cells (**Figure 2J**). Only the concentration of 100 nM simvastatin plus PLO did not show any difference in phase 1 secreted insulin at the 2.8 mM glucose challenge (**Figure 2J**). For pravastatin plus PLO treatment, the levels of secreted insulin during phase 1 were significantly less compared to PLO treatment alone in response to 16.8 mM of glucose challenge (**Figure 2K**, p<0.001), while only a high dose of pravastatin (10 μM) plus PLO caused a significant reduction of secreted insulin in response to 5.6 mM glucose, compared to control PLO alone treated cells (**Figure 2K**, p<0.01).

During phase 2 of insulin secretion, cells treated with PLO alone exhibited higher levels of secreted insulin compared to vehicle treated cells in response to 16.8 mM glucose challenge (**Figures 2L, M**, p<0.0001). Both simvastatin and pravastatin, at doses of 1 μM and 10 μM plus PLO, significantly reduced insulin secretion in response to 5.6 mM and 16.8 mM glucose challenge, compared to control PLO alone treated cells (**Figures 2L, M**, p<0.0001).

### 3.3 Effect of simvastatin and pravastatin treatments on mitochondrial function

**Figure 3:**
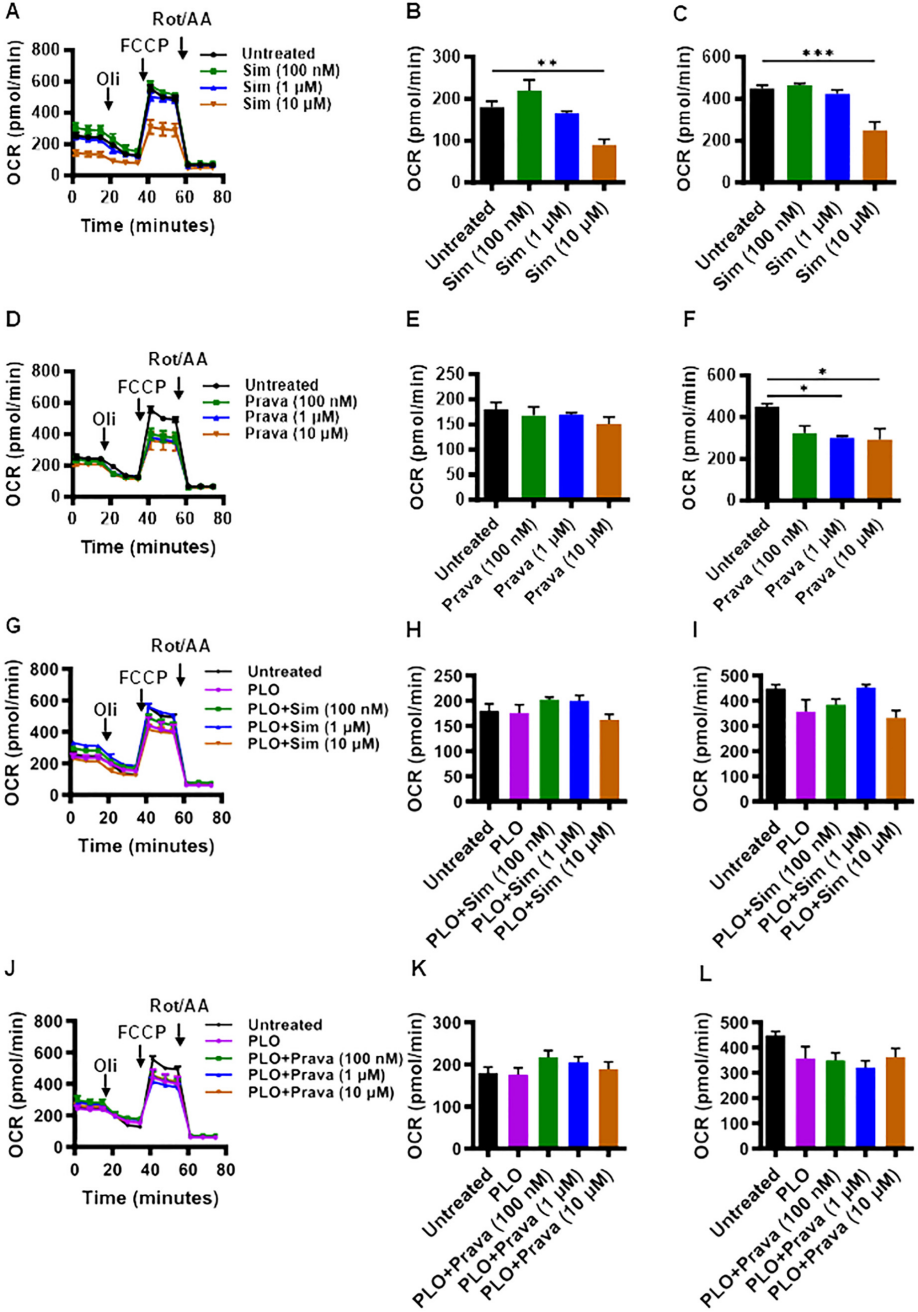
Effect of simvastatin and pravastatin on oxygen consumption rate (OCR) and mitochondrial respiration in MIN6 cells. MIN6 cells (4×10^4^/well) were cultured in 5.6 mM glucose concentration and were treated with different concentrations (100 nM; dark green color bars, 1 μM; blue color bars, and 10 μM; brown color bars) of simvastatin or pravastatin, alone or in combination with PLO (500 μM, purple color bars) for 72 hrs whereas the vehicle-treated cells served as untreated control (Black color bars). Cells were subsequently subjected to metabolic flux analysis for determination of oxygen consumption rate (OCR) using Seahorse assay as described in Materials and Methods. **(A)** OCR, **(B)** basal respiration, and **(C)** maximal respiration in cells treated with simvastatin alone. **(D)** OCR, **(E)** basal respiration, and **(F)** maximal respiration in cells treated with pravastatin alone. **(G)** OCR, **(H)** basal respiration, and **(I)** maximal respiration in cells treated with simvastatin plus PLO. **(J)** OCR, **(K)** basal respiration, and **(L)** maximal respiration in cells treated with pravastatin plus PLO. Each experiment was performed as at least 3 independent experiments, and each experiment was performed in triplicate. Group differences were analyzed using one-way analysis of variance (ANOVA) and p-values < 0.05 were considered statistically significant. Data are presented as Mean ± SEM; *p < 0.05; **p < 0.01; and ***p <0.001. Oil, Oligomycin; FCCP, Carbonyl cyanide 4-(trifluoromethoxy)phenylhydrazone; Rot/AA, Rotenone/Antimycin A.

Given the critical role of mitochondrial function in cell viability and mechanism of insulin secretion, we next asked whether treatments of MIN6 cells with simvastatin and pravastatin, alone or in combination with PLO, affected the mitochondrial function. To this end, metabolic flux analysis by seahorse assay revealed that at a concentration of 10 μM, simvastatin alone treatment led to significantly lower oxygen consumption (**Figure 3A**), with the reduced basal (**Figure 3B**, p<0.01) and maximal (**Figure 3C**, p<0.001) respirations, compared to vehicle treated (control) cells. Interestingly, pravastatin alone did not affect the basal respiration (**Figures 3D, E**), but it significantly reduced the level of maximal respiration at concentrations of 1 μM and 10 μM (**Figure 3F**, p<0.05). No significant changes in the basal and maximal respirations were observed as the cells were treated with a combination of simvastatin and PLO, compared to controls (vehicle-treated or treated with PLO alone) treatments (**Figures 3G–I**). No significant changes in the basal and maximal respirations were observed as the cells were treated with pravastatin plus PLO, compared to controls (**Figures 3J–L**).

### 3.4 Reversibility of suppressive effect of simvastatin on insulin secretion and lack of the effect of mevalonate replenishment on total insulin content

**Figure 4:**
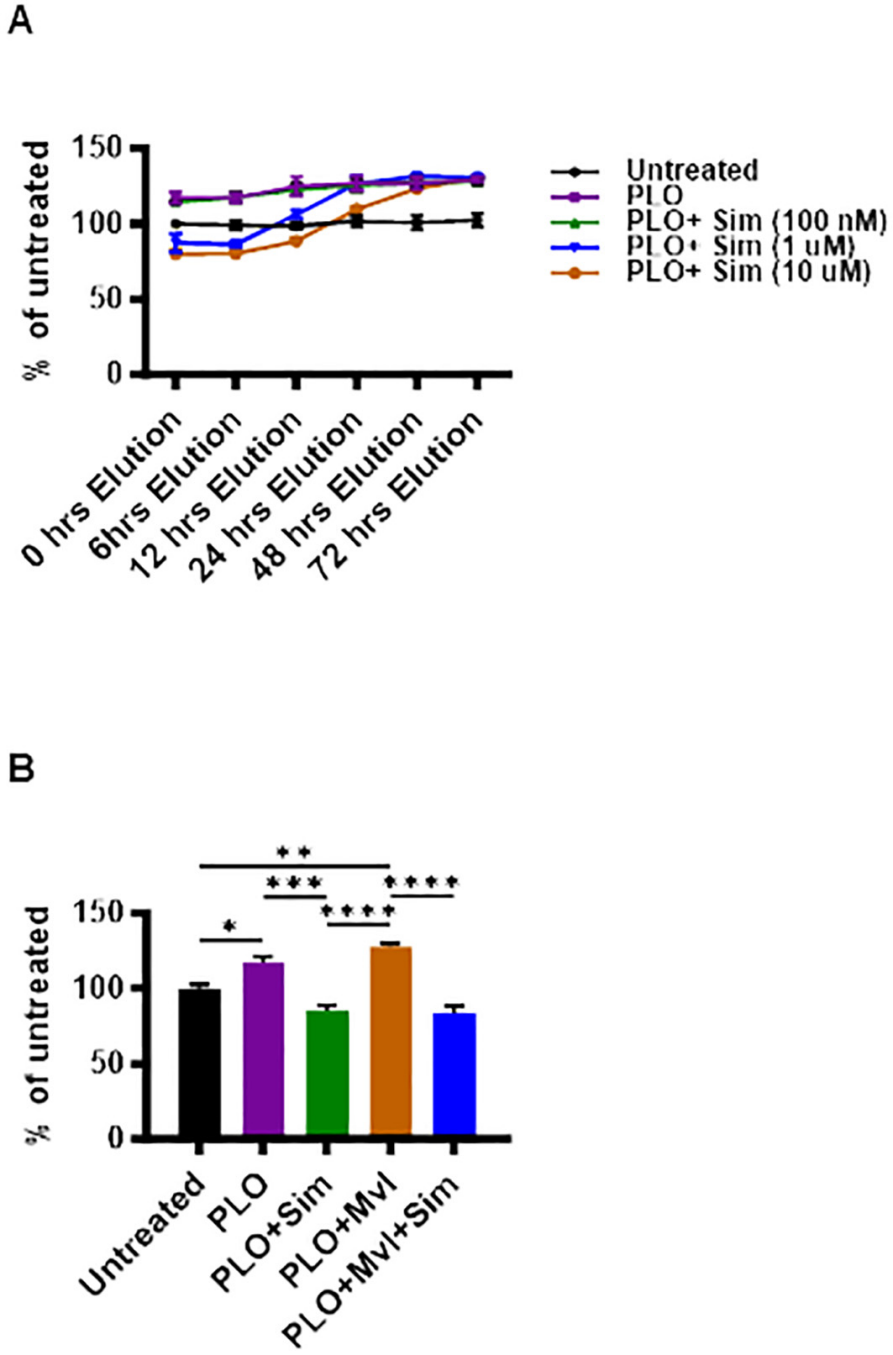
Reversibility of insulin-suppressive effect of simvastatin in PLO-treated MIN6 cells, with no effect of exogenous mevalonate on total insulin content. **(A)** MIN6 cells treated with different concentrations of simvastatin (100 nM; dark green color line, 1 μM; blue color line, and 10 μM; brown color line) in combination with PLO or PLO alone (500 μM, purple color line) for 72 hrs. Then the cells were PBS washed and fresh medium containing only the PLO was added. Total insulin content measured as described in Materials and Methods are shown at 6, 12, 24, 48, and 72 hrs; vehicle-treated cells representing untreated control (Black line). **(B)** MIN6 cells were treated with PLO alone (500 μM, purple bar), PLO plus simvastatin (10 μM; dark green bar), PLO plus mevalonate (1 mM; brown bar), PLO plus mevalonate plus simvastatin (blue bar), and vehicle-treated cells (Black bar) and total insulin content measured at 72 hrs are shown. Data are presented as a percentage ratio of the measured total insulin content in each condition over the total insulin content in untreated cells (control). Each experiment was performed as at least 3 independent experiments, and each experiment was performed in triplicate. Group differences were analyzed using one-way analysis of variance (ANOVA) and p values < 0.05 were considered significant. Data are presented as Mean ± SEM, *p < 0.05; **p < 0.01; ***p <0.001; and ****p <0.0001.

To investigate whether the suppression of insulin content in MIN6 cells following treatment with simvastatin plus PLO was transient, an elution experiment was conducted in which MIN6 cells were initially treated with simvastatin plus PLO for 72 hrs and then the media were refreshed with media lacking simvastatin only and total insulin contents were measured at different time points (6, 12, 24, 48 and 72 hrs). Notably, the insulin-suppressive effect of simvastatin on PLO-treated cells was transient and total insulin contents were restored during the washout period, in a dose-dependent manner (**Figure 4A**), such that the cells treated with a lower concentration of simvastatin (1 μM) plus PLO had insulin content restored at an earlier time point (24 hrs) as opposed to those treated with a higher concentration of simvastatin (10 μM) plus PLO which showed a relatively delayed insulin content recovery at 48 hrs.

In cholesterol biosynthesis pathway, mevalonate is produced by HMG-CoA, involving the enzymatic activity of HMG-CoA reductase. Given that both simvastatin and pravastatin are inhibitors of HMG-CoA reductase, we next tested the hypothesis whether replenishing mevalonate in our cell culture model could reinstate total insulin content in MIN6 cells treated with simvastatin (10 μM) plus PLO in the presence or absence of 1 mM of mevalonate after 72 hrs treatment. Although adding mevalonate to PLO significantly increased the total insulin content, compared to each of untreated, PLO alone, and simvastatin plus PLO treatment (**Figure 4B**, p< 0.01), its addition to cells challenged with simvastatin plus PLO failed to improve the total insulin content.

## 4 Discussion

Over the past few decades, several studies have been conducted using *in vitro* mouse and rat insulinoma cell culture models to investigate the various mechanisms of β-cell toxicity and function modulation, following the exposure to statins (25–33). However, statin effects were not assessed in the presence of free fatty acids which becomes important, given that these lipid-lowering agents are used to treat individuals suffering from dyslipidemia. In this study, we used mouse insulinoma MIN6 β-cell culture model to study the effects of simvastatin and pravastatin, in the presence of a physiologically relevant cocktail of free fatty acids including palmitic, linoleic and oleic acids (PLO). A clear correlation was found between the type of statin and the intensity of treatment with the development of T2D (20). Given the case that simvastatin has been reported as more diabetogenic than pravastatin in individuals with dyslipidemia (8, 12, 16, 17, 20, 22), we chose both of these drugs for this comparative study. Importantly, in this modified mouse β-cell culture model, we exposed the cells to statins for 72 hrs to emulate the biological effects of the long-standing chronic exposure to statin in real life therapy setting.

First, we demonstrated that in the absence of free fatty acids, simvastatin was toxic to MIN6 cells whereas pravastatin did not show any toxicity. These findings are corroborated by other studies as well (26–30). To minimize the confounding effects, the concentrations of statins (100 nM, 1 and 10 μM) and PLO (500 μM) used in this model were those that allowed a ≥80% cell viability. Interestingly, simvastatin toxicity was reduced in presence of PLO, which was an unexpected finding. In our model, simvastatin was found to be more effective in lowering total intracellular cholesterol content compared to pravastatin which may not be surprising, given that an open, randomized, parallel comparative study of simvastatin and pravastatin, involving 100 patients with primary hypercholesterolemia and using the recommended starting dose, reported a significantly greater lipid-lowering effect of simvastatin compared to pravastatin (40).

Notably, we found that the addition of PLO significantly increased the intracellular cholesterol content which might explain the reduced toxicity of simvastatin for cells in the setting when PLO was added to the medium. It is well known that cholesterol is an important component of cell membrane which contributes to cell survival and overall regulation of biophysical properties of cell membrane such as fluidity, biomolecular transport, and vesicle fusion in β-cells (41).

Next, we looked at the effects on β-cell function following exposure to simvastatin and pravastatin, in presence and absence of PLO by measuring total insulin content, GSIS, as well as phase 1 and phase 2 insulin secretion. We found that the addition of PLO significantly increased the level of total insulin content in the cells. Furthermore, simvastatin but not pravastatin significantly reduced the level of total insulin content at higher doses of 1 and 10 μM. The GSIS results show that both simvastatin and pravastatin reduced the secreted insulin in response to glucose challenge at a concentration of 16.8 mM. However, only simvastatin treatment in presence of PLO was able to suppress insulin secretion at a glucose challenge of 5.6 mM, compared to PLO alone treatment. It is important to mention that in our MIN6 cell culture model, insulin secretions were not affected by simvastatin and pravastatin treatments, whether in presence or absence of PLO, in response to challenge by KCl, implying that statin effects might be mediated through mechanisms involving glucose sensing and insulin secretory machinery. However, unlike our findings, a previous study using MIN6 cells reported a decreased KCl-induced insulin secretion (28), which may be attributed to differences between two studies regarding concentrations of simvastatin and KCl, duration of exposure to statins, and more importantly, the lack of lipids in culture model used in previous study.

Notably, insulin is released from β-cells in a bi-phasic pattern, called phase 1 and phase 2, in response to glucose challenge. Phase 1 insulin secretion is defined as a brief spike lasting for about 10-15 min releasing the previously synthesized and stored insulin, followed by phase 2 which reaches the plateau at 2 hrs and represents the new insulin production and secretion (42, 43). The GSIS response accounts for measuring insulin secretion from β-cells during both phases 1 and 2 (43). To this effect, we next investigated whether simvastatin and pravastatin affected insulin secretion at both phases. We found that both simvastatin and pravastatin suppressed the insulin secretory responses in a dose-dependent manner, during phase 1 as well as phase 2, a finding which has not been reported before to the best of our knowledge.

Addressing insulin secretory mechanism of β-cells, the role of glucose in oxidative metabolic pathways is well established (44, 45). Following glucose sensing, the increase in ATP/ADP ratio blocks the ATP-sensitive K^+^ channels (K-ATP), causing depolarization of the membrane potential (25, 46, 47) and activation of L-type voltage-gated Ca^2+^ channels (VGCCs) (48), resulting in insulin vesicle exocytosis. It was reported that simvastatin impaired mitochondrial respiration and ATP production in β-cells that were treated with simvastatin (29, 32). Simvastatin reduced the cytosolic ATP/ADP ratio and inhibited OCR (25, 46). In these studies, although much higher concentrations (1-10 mM) of simvastatin were used compared to the concentrations used in our study, we still debated if MIN6 cells exposure to simvastatin and pravastatin could modulate or impair the mitochondrial respiration in these cells. Our OCR findings reveal that higher concentration of simvastatin (10 μM) reduced both the basal and maximal respirations while pravastatin (1 and 10 μM) reduced only the maximal respiration. Since simvastatin was found to suppress mitochondrial respiration at both the basal and maximal levels, this likely accounts for its greater effect on insulin suppression, compared to pravastatin. Our results showing a stronger effect of simvastatin than pravastatin on mitochondrial respiration is corroborated by other studies, supporting that simvastatin (a lipophilic statin) affected the mitochondrial function more as compared to pravastatin (a hydrophilic statin), with stronger effects on the inner mitochondrial membrane (49) and disruption of membrane protein-lipid interactions (50). However, we found that in presence of PLO, no significant changes in mitochondrial respiration were observed for both simvastatin and pravastatin.

Statins are the competitive inhibitors of HMG-CoA reductase which catalyzes the rate limiting reaction regulating conversion of HMG-CoA to mevalonate in cholesterol biosynthesis pathway (51, 52). Our results from elution experiments indicate that MIN6 β-cells were able to restore total insulin content within 48 hrs of simvastatin withdrawal, compared to PLO treatment as a control, while requiring longer recover times for high drug concentrations. This reversibility of changes in β-cell function (total insulin content) induced by simvastatin is highly important, implying that a therapy withdrawal or change of dyslipidemia medications might warrant the prevention of new-onset T2D or the remission of statin-induced diabetes in clinical settings. Moreover, we found that adding mevalonate exogenously to the culture medium containing simvastatin plus PLO did not improve total insulin content. Indeed, another study also found that the addition of mevalonate could not restore MIN6 β-cell function in terms of insulin secretion (28).

Despite the risk for new-onset T2D, statins remain the first-choice medications for patients with established clinical atherosclerotic disease or at high risk of it, for patients with diabetes aged 40-75, and for those with highly elevated LDL ≥190 mg/dL. This is well justified based on a favorable risk-benefit ratio for clinical practice. However, advent of the new generation of lipid-lowering drugs is paving way for switching from mono-therapeutic to poly-therapeutic treatment options (53).

Nonetheless, this study has certain limitations involved, such as in-depth molecular mechanisms and the pathways of insulin biosynthesis and maturation, ATP/ADP ratios, Ca^2+^ flux, miochondrial ion channels and membrane potential changes remain to be elucidated.

Although our study provides valuable insights into the effect of statins on mouse MIN6 β cell line, it involves certain limitations viz we have used a single cell line (MIN6 cells) in this study and it would be interesting to also compare the effects of simvastatin and pravastatin on other β-cell models, such as human EndoC-βH1 cell line, in future studies to further verify and strengthen the data presented in this study.

## 5 Conclusion

Taken together, using our modified MIN6-PLO mouse β-cell culture model, we identified that chronic exposure to simvastatin induced a more proficient cholesterol lowering, albeit with a higher β-cell toxicity as compared to pravastatin. Of note, the suppression in insulin secretion was induced only by simvastatin, while both drugs did not impair the mitochondrial respiration. The undesirable effects of simvastatin on β-cell viability and insulin secretion are attributed to its lipophilic nature, allowing it to diffuse easily into cells, whereas pravastatin requires the presence of active protein transporters that are mainly found in the liver. Importantly, the total insulin content suppression by simvastatin was found to be reversible, pointing to the rescue potential of simvastatin-induced untoward changes in MIN6 β-cells. Future studies may extrapolate this work by investigating β-cell survival and function changes in response to new therapeutic approaches such as the combined use of proprotein convertase subtilisin/kexin type 9 (PCSK9) inhibitors and statins for treating hyperlipidemia.

## Data Availability

The original contributions presented in the study are included in the article/**Supplementary Material**, further inquiries can be directed to the corresponding author/s.
