# Supplementary material for: Comparative efficacy, toxicity, and insulin-suppressive effects of simvastatin and pravastatin in fatty acid-challenged mouse insulinoma MIN6 β-cell model
Source: Front Endocrinol (Lausanne). 2024 Oct 31;15:1383448. doi: 10.3389/fendo.2024.1383448 (PMC11560436; doi:10.3389/fendo.2024.1383448)
Supplement: Supplementary file 1 [file DataSheet1.pdf]

Supplementary Figure: S1

Title of the manuscript: Comparative efficacy, toxicity, and insulin-suppressive effects of simvastatin and pravastatin in fatty acid-challenged mouse insulinoma MIN6 β-cell model

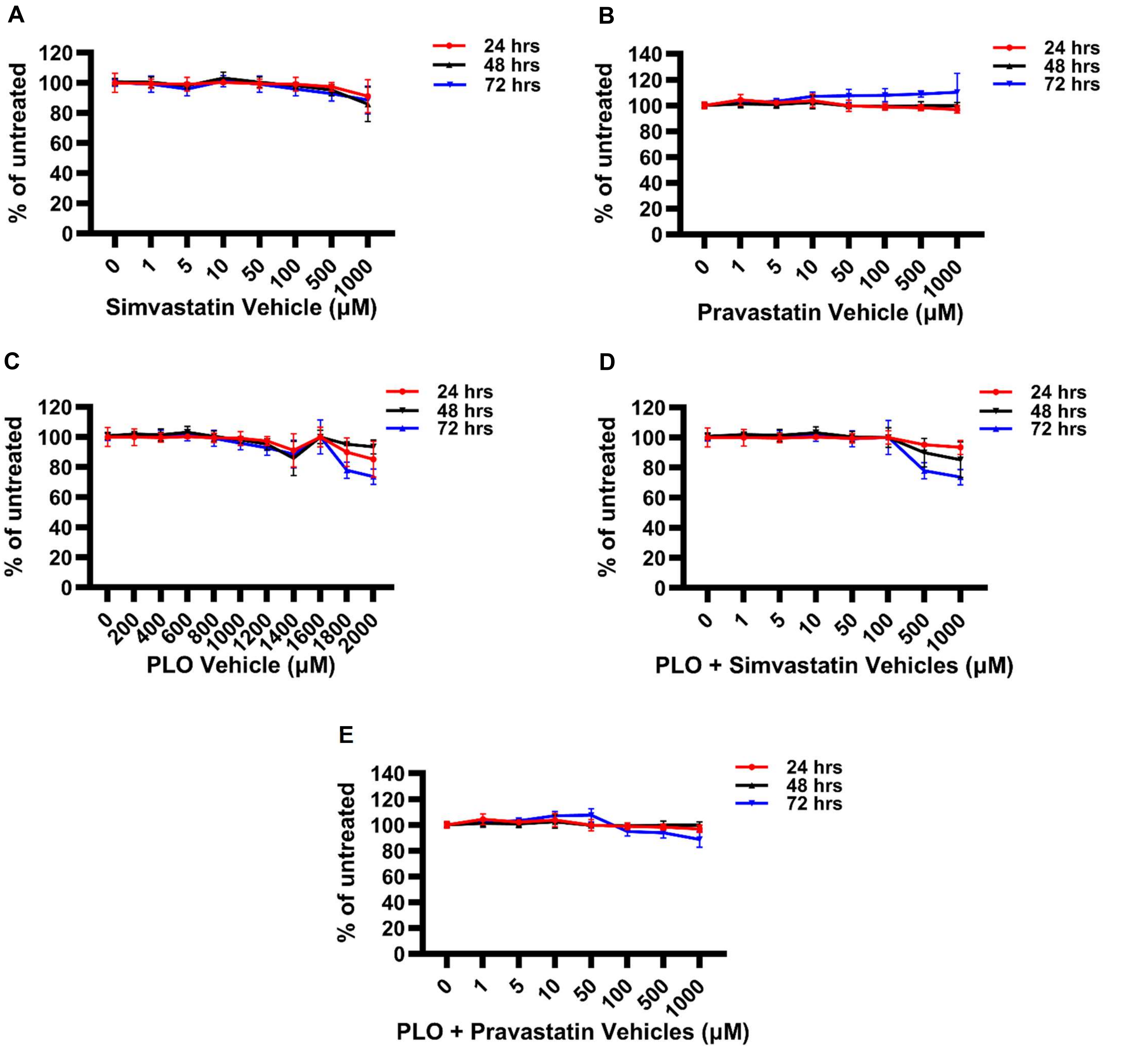

SUPPLEMNETARY FIGURE S1 Toxicity effects of simvastatin, pravastatin, and PLO vehicles in mouse insulinoma MIN6 cells. MIN6 cells were maintained in supplemented DMEM media with 5.6 mM glucose and were treated with different concentrations of either (A) ethanol as simvastatin vehicle or (B) PBS as pravastatin vehicle, or (C) BSA and ethanol as PLO vehicle for 24 hrs (red line), 48 hrs (black line) and 72 hrs (blue line). In our modified MIN6-PLO set-up, MIN6 cells cultured in 5.6 mM glucose were treated with a combination of PLO vehicle and with either (D) simvastatin or (E) pravastatin vehicles for 24 hrs (red line), 48 hrs (black line) and 72 hrs (blue line). MTT assay was conducted to measure the lethal dose of 50% survival (LD_{50}). Data are presented as mean ± SEM values from n = 3 independent experiments, each experiment was done in triplicate.
